# Effects of High-Intensity Swimming Interval Training on Area, Perimeter, Circularity Index and Phenotype of Cardiac Mitochondrial Ultrastructure in Sprague Dawley Rats

**DOI:** 10.3390/life14080984

**Published:** 2024-08-06

**Authors:** Grace Pasmiño, Marco Paredes, Héctor Silva

**Affiliations:** 1 Programa de Doctorado en Ciencias Morfológicas, Facultad de Medicina, Universidad de La Frontera, Temuco 4811230, Chile; g.pasmino01@ufromail.cl; 2 Laboratorio Fisiología del Ejercicio, Departamento de Ciencias Básicas, Facultad de Medicina, Universidad de La Frontera, Temuco 4811230, Chile; 3 Laboratorio de Biología Celular, Departamento de Ciencias Básicas, Facultad de Medicina, Universidad de La Frontera, Temuco 4811230, Chile; marco.paredes@ufrontera.cl

**Keywords:** heart, mitochondria, ultrastructure, morphology, transmission electron microscopy, high-intensity interval training, exercise, sedentary lifestyle, prevention, adolescent

## Abstract

Physical inactivity impairs health by increasing morbidity. In childhood, modifiable risk factors associated with cardiovascular pathologies and related to mitochondrial function and structure are initiated by physical inactivity. The objective of this study was to analyze the effect of high-intensity swimming interval training (HIIT-swim) on cardiac mitochondrial ultrastructure in young Sprague Dawley rats compared with a sedentary group. Five-week-old Sprague Dawley rats (n = 18) were divided into a control group (C) (n = 6), a sedentary group (S) (n = 6) and an HIIT-swim group (H-s) (n = 6), the last of which performed HIIT-swim for 4 weeks. A mitochondrial ultrastructural evaluation was performed using transmission electron microscopy. In the H-s rats, mitochondrial areas and perimeters were found to be statistically significantly different from those of the C and S rats. In addition, no predominant intramitochondrial multifragmentation was observed in the mitochondria of H-s rats, but multifragmentation was evident in the mitochondria of S rats.

## 1. Introduction

During childhood, modifiable risk factors affecting cardiovascular pathologies are known to emerge [1]. Cardiovascular and respiratory capacity both increase during adolescence and early adulthood but may then decline due to a lack of physical activity [2]. Individuals who do not engage in physical activity face a higher level of coronary risk than those who regularly exercise [3,4].

The combined capacity of an individual’s cardiac and respiratory systems during youth may be associated with the cardiometabolic health status of that individual later in life; such an outcome is in line with the cross-sectional association between physical fitness and cardiometabolic health status in childhood and adolescence [5]. Early intervention, including the adoption of prevention strategies such as exercise and physical training activities [6] that promote heart health, can help children maintain or achieve a healthy state; by such means, many future health problems may be prevented.

Poor cardiovascular fitness is associated with an elevated risk of cardiovascular disease [7]. However, it takes a long time for such a disease to develop as a result of physical inactivity, and there are no obvious clinical symptoms in the early stages [8]. Therefore, the risk factors for cardiometabolic diseases begin in childhood, and the likelihood of these factors being retained throughout life increases from childhood onwards [9].

Physical inactivity negatively impacts health and increases both morbidity and mortality; this, in turn, increases the financial burden on public health services [10]. In contrast, long-term physical training improves quality of life and decreases the risk of hospitalization and mortality [11]. It is estimated that more than 80% of adolescents do not meet the physical activity levels recommended by WHO [10]. 

Cardiovascular pathologies, as well as other diseases such as obesity and metabolic disorders, are related to the function and structure of mitochondria [12,13,14]. Mitochondria are essential for the high energy requirements of the heart [15] and are estimated to account for 40% of cardiomyocyte volume [16]. Mitochondria have the capacity to precisely meet the energy needs of myocytes [17] and reflect the capacity of the heart to perform physical work and to produce energy [18].

Loss of regulatory mechanisms and mitophagy result in decreased efficiency in energy production and increased dysfunctional mitochondria [19] along with alterations in mitochondrial structure due to the dysfunction of quality control mechanisms during cardiac diseases [16]. With declining cardiac fitness, the premature onset of chronic pathologies may occur, increasing the risk of mortality in adult life stages [20]. 

Mitochondrial dynamics are reflected in the structure of mitochondria, and mitochondria can present with heterogeneous shapes and sizes as a result of physiological changes. The impact of morphological variations is reflected in the cellular metabolism [21]. Therefore, an evaluation and analysis of morphometric and qualitative parameters allows a more comprehensive analysis of the functional state of mitochondria and of any relationship with changes in the mitochondrial ultrastructure [22,23].

Alterations in the mitochondrial structure are related to the loss of regulatory mechanisms involved in mitophagy, resulting in the accumulation of dysfunctional mitochondria [13,19]. In contrast, a classical mitochondrion indicates functional homeostasis. Increased numbers of mitochondria in the heart, with multiple points of interrupted mitochondrial crests, have been associated with alterations in energy productivity and have also been related to alignment patterns [24,25]. A structural transition related to the progression of ridge curvatures has been observed in progressive age course-dependent changes [26], as well as mitochondria with phenotypic characteristics similar to those of defective cardiomyocytes [27]. 

Resistance training has been shown to reverse the ultrastructural morphology of diabetic cardiomyopathy, leading to nondiabetic phenotypes [28,29]. Such training has also been reported to improve cardiovascular function and insulin signaling [11,19]. Aerobic-exercise training increases cardiorespiratory fitness levels, significantly lowering the risk of cardiovascular disease mortality reported for the general population [3].

In addition, high-intensity interval training (HIIT) is a cardioprotective factor that counteracts metabolic dysregulation [30]. HIIT induces effects on the left ventricle, improving both diastolic and systolic function [31,32], due to decreased systemic vascular resistance and/or increased myocardial contractility [31]. This significantly improves the cardiac ejection fraction [32].

The aim of this research was to evaluate the effect of high-intensity swimming interval training (HIIT-swim) on the ultrastructures of heart mitochondria in young (adolescent) Sprague Dawley rats compared with sedentary rats.

The rats were not used as a model for the assessment of aging nor were they used transgenically for assessment of cardiovascular pathology or pathophysiological stress; the study objective was simply to evaluate the mitochondria in HIIT-swim and healthy sedentary rats.

## 2. Materials and Methods

### 2.1. Ethical Considerations

The research protocol was approved by the Scientific Ethical Committee of La Universidad de La Frontera (N° 063_21). Throughout the process, animal welfare was prioritized, and the guidelines set out in the National Institutes of Health Guide for the Care and Use of Laboratory Animals [33] were followed. In addition, the monitoring protocol for rodents by Morton and Griffin [34] was applied.

### 2.2. Animal Model 

For this study, 18 5-week-old young (adolescent) male rats of the Sprague Dawley strain (*Rattus norvegicus*) were used. The rats were maintained in the Basic Sciences Biotherium of the Universidad de La Frontera under the care of a Biotherium veterinarian with special expertise in the care and maintenance of research animals. The animals were kept in a temperature-controlled room at 25 ± 2 °C with a 12 h light/12 h dark cycle and were provided with food and water ad libitum. The cages did not contain exercise wheels, mazes or anything else that would promote physical activity in the rats.

**Figure 1:**
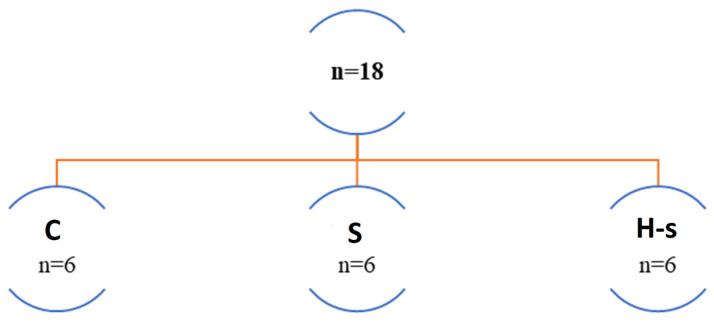
Distribution of research groups. C—control group; S—sedentary group; and H-s—HIIT-swim group.

The animals were randomly divided into the following three groups: a control group (C, n = 6), a sedentary group (S, n = 6) and a HIIT-swim group (H-s, n = 6) (Figure 1). 

C rats were killed at 5 wk of age. H-s rats were started on the 4-week HIIT-swim protocol at 5 wk of age [35] and were killed at 9 wk of age. The 5-week-old S rats, which were kept in a sedentary state while the H-s rats performed the training protocol, were killed at 9 wk of age at the end of the H-s rats’ 4 wk protocol.

Rats were killed with an injection of sodium pentobarbital by the Biotherium veterinarian, according to the protocol approved by the Scientific Ethical Committee of La Universidad de La Frontera (N° 063_21).

### 2.3. High-Intensity Swimming Interval Training (HIIT-Swim) Protocol

The research pool was 160 cm in diameter and 80 cm in height [36]. It was filled with water to a depth of 40 cm and maintained at a constant temperature of 32 ± 2 °C. 

The H-s rats were allowed a period of adaptation to the water-filled pool. This involved daily 15 min periods in the pool, without weights, for 5 consecutive days. 

During resting periods, the rats were gently dried with towels and kept close to the investigator so that they could get to know him and adapt to both his presence and the laboratory; they were subsequently returned to their cages. In addition, the laboratory was equipped with fan heaters that maintained an adequate room temperature; this ensured optimal drying conditions and promoted the well-being of the rats.

Over the course of the 4 wk training period, the HIIT-swim protocol was performed on alternate days in the exercise physiology laboratory. The reference protocol proposed by Amirazodi et al. [35] was used, consisting of 14 sets of 20 s swimming periods followed by 10 s rest windows. At the end of each session of the HIIT-swim protocol, the parameters of the monitoring protocol for rodents by Morton and Griffin [34] were checked.

### 2.4. Transmission Electron Microscopy Protocol

Cardiac muscle tissue was obtained after the rats were killed; samples were systematically, uniformly and randomly selected [37,38]. The tissues were then processed for resin embedding.

Samples were fixed with a solution of 4% paraformaldehyde and 2.5% glutaraldehyde. Three washes, each of 10 min duration, were then performed using 0.1 M cacodylate buffer with a pH of 7.2. This was followed by post-fixation with 1% osmium tetroxide in 0.1 M cacodylate buffer for 2 h at 4 °C. Samples were then washed with distilled water. Subsequently, 2% uranyl acetate was added. Samples were left under the hood for 1 h and then washed with distilled water. 

Next, the samples were dehydrated in an increasing battery of ethanol (30º, 50º, 70º, 95º, 100º and 100º) and acetone 1 and 2 for 15 min each; they were then placed in a mixture of acetone and resin (1:1) overnight under stirring at room temperature. 

Subsequently, the mixture was removed and new pure resin added. Samples were left in an oven with a vacuum for 8 h at room temperature; after this time, the resin was removed and new resin was added to enable polymerization in the course of a 24 h period in a 60 °C oven.

Semi-thin sections of 500 nm were stained with toluidine blue, and ultrathin sections of 80 nm were then obtained on a 300 mesh copper grid; these were stained with 4% uranyl acetate in methanol for 2 min and then added to lead citrate for 5 min. 

The cuts were performed with a Leica EM UC7 ultramicrotome. The samples were observed under a Zeiss Libra 120 electron microscope at 120.000 Kv.

### 2.5. Mitochondrial Ultrastructural Evaluation of the Cardiac Muscle

A total of 1028 mitochondria were morphometrically analyzed as follows: 333 from the C group, 358 from the S group and 337 from the H-s group. The parameters used for this analysis were estimates of the size (perimeter and area) and shape (circularity index) of the mitochondria [39].

The circularity index values ranged between 0.0 (low value) and 1.0 (high value). A value close to 0.0 indicated an elongated shape, and as values increased towards 1.0, a shape closer to that of a perfect circle was indicated [40].

A morphometric evaluation was then performed using Fiji software version 2.9.0 [41]. First, for each image obtained using transmission electron microscopy, normalization and registration of the measurement scale was carried out. Manual selection of each mitochondrion was then carried out using the polygon selections tool. Using the measured tool, quantification was carried out and the values of the mentioned parameters were recorded.

### 2.6. Statistical Analysis

GraphPad Prism 9 software version 9.5.1 (San Diego, CA, USA) was used to perform the statistical analysis. For data analysis, the Kruskal–Wallis test was used, followed by Dunn’s test. To determine the difference between any two groups, a t-test was used. The data are presented as the mean (M), and the standard error of the mean is expressed as ± SEM. All test results with *p*-values < 0.05 and 95% confidence intervals were considered statistically significant.

## 3. Results

### 3.1. Mitochondrial Morphological Parameters

The results of the quantification of the mitochondrial morphological parameters of C, S and H-s are shown in Table 1.

The circularity indexes of S and H-s rats were found to differ significantly from those of rats in the C group. In addition, the mitochondrial areas and perimeters of H-s rats were significantly different from those of C and S rats. Finally, the aforementioned parameters were found to be significantly different in the S rats compared with the C rats.

#### 3.1.1. Mitochondrial Shape: Mitochondrial Circularity Index

**Figure 2:**
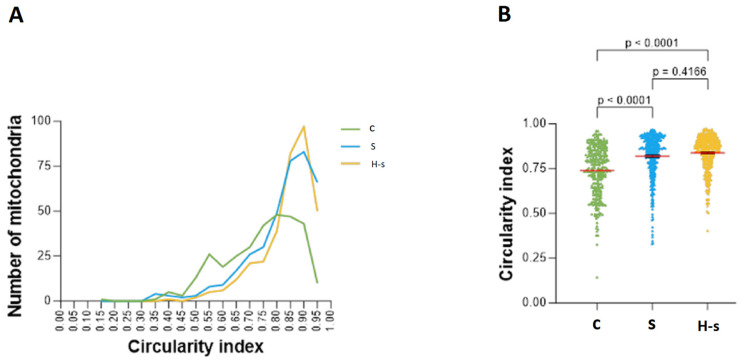
Mitochondrial circularity indexes for C, S and H-s rats. (**A**) Representative graph of the frequencies of the number of mitochondria. (**B**) Scatter plot of the circularity index of mitochondria. The mean of each group is represented by a red horizontal line. *p* < 0.05 was considered statistically significant.

A total of 97 of the 337 mitochondria analyzed from the H-s [28.8%] rats had a circularity index between 0.9 and 0.95; 48 of the 333 mitochondria analyzed from the C [14.4%] rats had a circularity index between 0.8 and 0.85; finally, 83 of the 358 mitochondria analyzed from the S rats [23.2%] had a circularity index between 0.9 and 0.95 (Figure 2A).

Average values for the circularity indexes of H-s, S and C mitochondria were 0.837, 0.818 and 0.738, respectively, indicating significant differences between C and H-s mitochondria and C and S mitochondria (*p* < 0.0001; Figure 2B). While mitochondria with high circularity indexes were mainly found in heart muscle tissue from older rats (H-s, S), younger rats (C) exhibited both elongated mitochondria (low index) and perfectly round mitochondria (high index) (Figure 2B).

#### 3.1.2. Mitochondrial Size: Area and Perimeter

##### Mitochondrial Area

**Figure 3:**
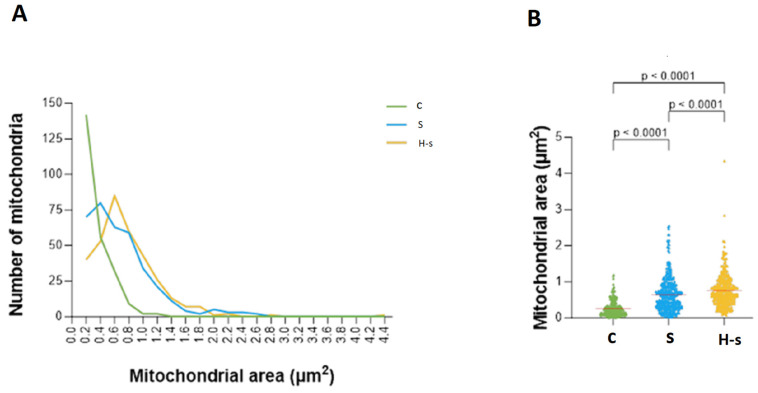
Mitochondrial areas of C, S and H-s rats. (**A**) Representative graph of mitochondrial area frequencies. (**B**) Scatter plot of mitochondrial area. The mean of each group is represented by a red horizontal line. *p* < 0.05 was considered statistically significant.

A total of 85 of the 337 mitochondria analyzed from the H-s rats [25.2%] had an area between 0.6 and 0.8 µm^2^; 142 of the 333 mitochondria analyzed from the C rats [42.6%] had an area between 0.2 and 0.4 µm^2^; finally, 80 of the 358 mitochondria analyzed from the S rats [22.3%] had an area between 0.4 and 0.6 µm^2^ (Figure 3A).

Average values for the areas of H-s, S and C mitochondria were 0.761, 0.645 and 0.262 µm^2^, respectively, indicating significant differences between C and H-s mitochondria and C and S mitochondria (*p* < 0.0001; Figure 3B). While mitochondria with larger areas were mainly found in heart muscle tissue from older rats (H-s, S), mitochondria from younger rats (C) showed smaller areas (Figure 3B).

##### Mitochondrial Perimeter

**Figure 4:**
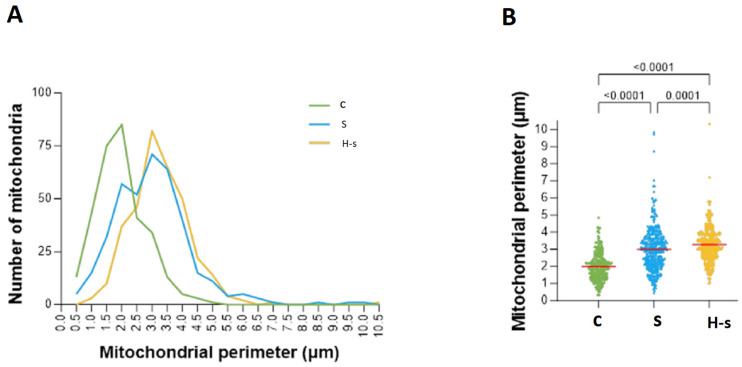
Mitochondrial perimeters of C, S and H-s rats. (**A**) Representative graph of mitochondrial perimeter frequencies. (**B**) Scatter plot of mitochondrial perimeter. The mean of each group is represented by a red horizontal line. *p* < 0.05 was considered statistically significant.

A total of 82 of the 337 mitochondria analyzed from the H-s rats [24.3%] had a perimeter between 3 and 3.5 µm; 85 of the 333 mitochondria analyzed from the C rats [25.5%] had a perimeter between 2 and 2.5 µm; finally, 71 of the 358 mitochondria analyzed from the S rats [19.8%] had a perimeter between 3 and 3.5 µm (Figure 4A).

Average values for the perimeters of H-s, S and C mitochondria were 3.264, 2.992 and 1.982 µm, respectively, indicating significant differences between C and H-s mitochondria and C and S mitochondria (*p* < 0.0001; Figure 4B). While mitochondria with higher perimeters were mainly found in heart muscle tissue from older rats (H-s, S), mitochondria from younger rats (C) exhibited lower perimeters (Figure 4B).

These results showed that HIIT-swim induced significant increases in mean area and girth in H-s rats compared with S (sedentary) rats. The results also showed nonsignificant variations in the circularity index, indicating conservation of the mitochondrial shape. Overall, these results suggested optimization and conservation of cardiac mitochondrial function by relating structure to function [23]. HIIT-swim led to a significantly increased mitochondria area (*p* < 0.0001; Figure 3B) and a significantly increased mitochondria perimeter (*p* < 0.0001; Figure 4B). Both groups were of the same age and had access to water and food ad libitum.

### 3.2. Transmission Electron Microscopy

**Figure 5:**
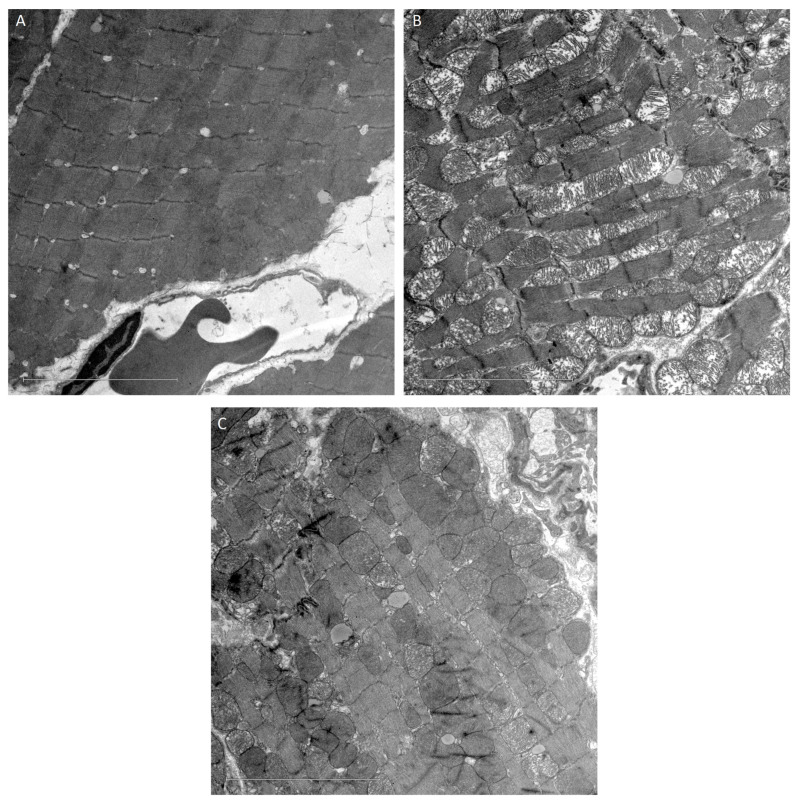
A comparative set of transmission electron microscopy images taken from C, S and H-s rats. Cardiac mitochondrial ultrastructure. ×2000. Scale bar = 5 µm. (**A**) Control group (C). (**B**) Sedentary group (S). (**C**) HIIT-swim group (H-s).

Transmission electron microscopy was used to observe and analyze the effect of the HIIT-swim protocol on cardiac mitochondrial ultrastructure in H-s rats compared with animals in the C and S groups. A comparative set of images from C, S and H-s rats is presented in Figure 5.

Age-related remodeling of mitochondria in cardiac tissue was lower in tissues from 9-week-old HIIT-swim (H-s) rats (Figure 5C) than in tissues from 9-week-old S rats (Figure 5B) when these were compared with tissues from 5-week-old C rats (Figure 5A).

When comparing the 5-week-old C rats with the 9-week-old S rats, contrasting electrodensities were observed as a result of the internal structural organization of the C mitochondria (Figure 5A) and the morphological transitions observed in the S mitochondria (Figure 5B) with respect to both mitochondrial ridge arrangement and mitochondrial ridge fragmentation.

**Figure 6:**
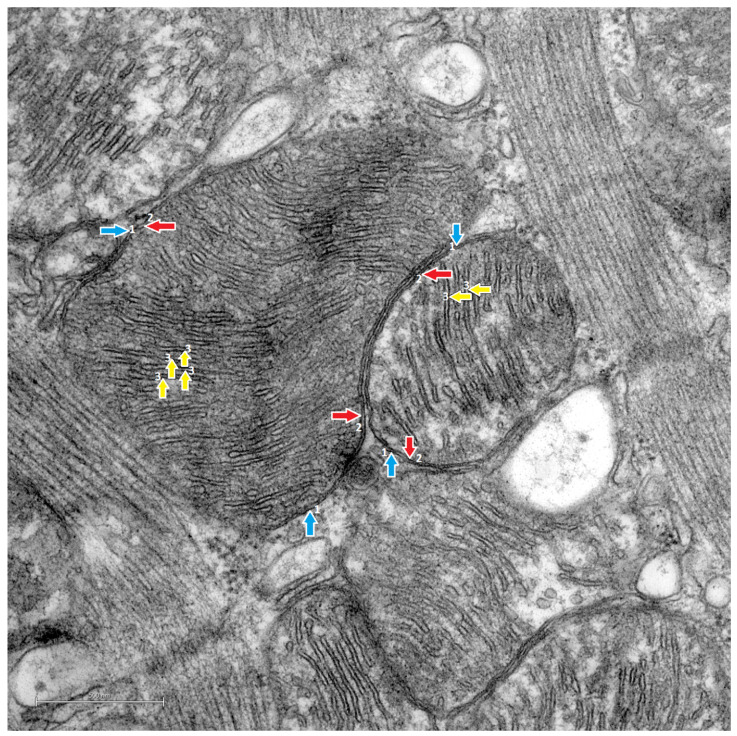
Transmission electron microscopy images. Cardiac mitochondrial ultrastructure of the H-s rats. ×10,000. Scale bar = 500 nm. The 5-week-old H-s rats performed the HIIT-swim protocol for a period of 4 weeks, ending at 9 weeks of age. In the image, the most relevant and representative ultrastructural observations of the mitochondrial phenotypes of the H-s rats are highlighted with arrows. The outer mitochondrial membrane (1, blue arrow), inner mitochondrial membrane (2, red arrow) and parallel mitochondrial ridges (3, yellow arrow) were observed.

**Figure 7:**
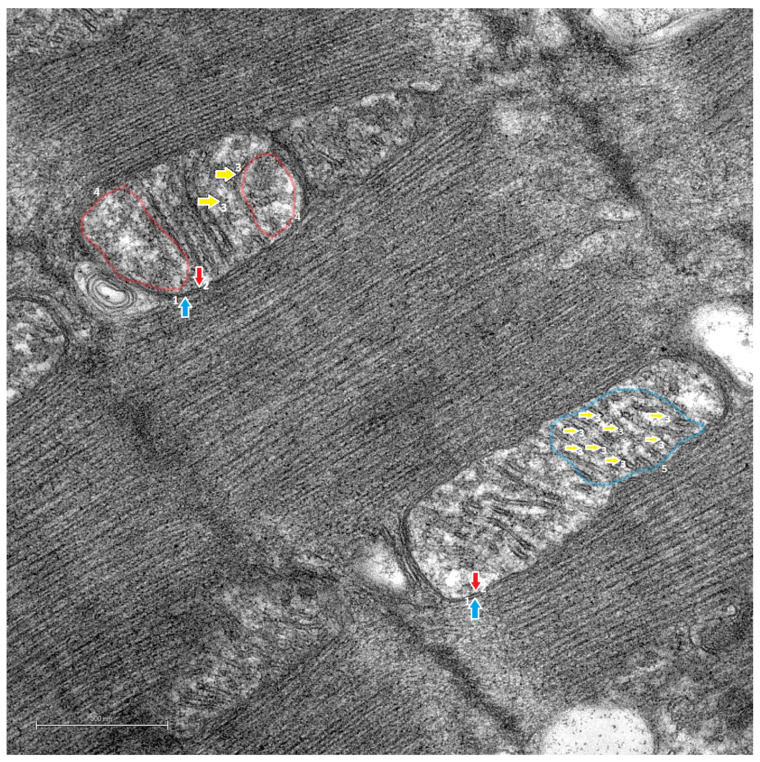
Transmission electron microscopy images. Cardiac mitochondrial ultrastructure of the S rats. ×10,000. Scale bar = 500 nm. The 9-week-old S rats did not perform the HIIT-swim protocol and were kept in a sedentary state without exposure to any physical activity-promoting elements such as wheels or physical activity mazes. The image shows the representative mitochondrial ultrastructure of the S rats, with the outer mitochondrial membrane (1, blue arrow), inner mitochondrial membrane (2, red arrow) and mitochondrial cristae (3, yellow arrow). A translucent red outline (4) demarcation indicates the representative areas of the ground-glass mitochondria, and a light blue outline (5) indicates a representative area of multifragmented mitochondrial cristae.

**Figure 8:**
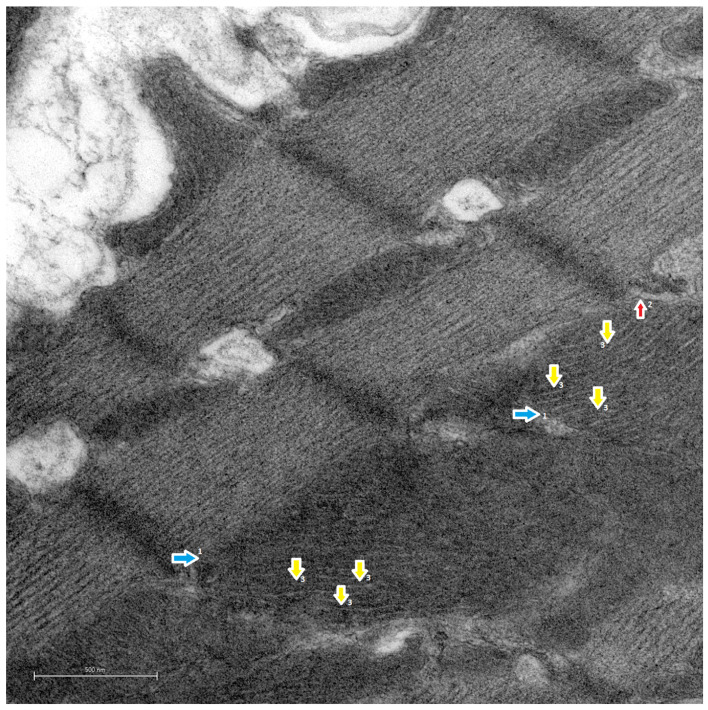
Transmission electron microscopy images. Cardiac mitochondrial ultrastructure of the C rats. ×10,000. Scale bar = 500 nm. The 5-week-old C rats were killed prior to the initiation of the HIIT protocol and were not exposed to any physical activity-promoting elements such as wheels or physical activity mazes. Representative observations of the C mitochondria are indicated with arrows. Outer mitochondrial membrane (1, blue arrow), inner mitochondrial membrane (2, red arrow) and mitochondrial crests (3, yellow arrow).

Higher-magnification images of mitochondria from H-s (Figure 6), S (Figure 7) and C (Figure 8) rats revealed further noteworthy results. For example, in the H-s rats (Figure 6) that were subjected to the HIIT-swim protocol, it was observed that the outer mitochondrial membrane delimited the mitochondria, while the inner membrane was organized in ridges with little fragmentation.

Specifically, the mitochondrial ridges were arranged in parallel and were located in close proximity to each other. This means that the cristae occupied a larger part of the internal space of the mitochondria compared with the S rats (Figure 7). 

In addition, a mitochondrial phenotype with a less elongated and more rounded shape was observed.

Mitochondria from S rats (Figure 7) showed an ultrastructure with internal membranes resulting in fragmented mitochondrial ridges at several points and, in some cases, loss of their parallel alignment. In addition, sectors in the mitochondria were observed to have a translucent and disrupted appearance due to the loss of density caused by the multifragmentation of the ridges, as well as a reduced proximity and a reduced parallel alignment, compared with mitochondria from H-s rats. 

In S rats, the mitochondria were observed to have internal areas with a frosted glass-like appearance, as well as fragmented crests.

In C rats (Figure 8), the inner membrane of the mitochondrion was characterized by the formation of densely parallel mitochondrial ridges, which occupied most of the intramitochondrial space. This gave the mitochondrion a more electrodense appearance upon observation. Thus, no disorganization, disruption or multiple internal fragmentations were observed.

In the present study, when comparing the morphological characteristics of the mitochondrial cristae of the H-s rats (Figure 6) with those of S rats (Figure 7), differences in morphology were observed. In other studies, remodeling of the cristae due to changes in the respiratory and functional states of the mitochondria has been observed [42]; consequently, remodeling in the form of structural variations could be associated with functional changes [43]. Therefore, the structural variations in the H-s cristae could correspond to cristae remodeling, probably as an effect of HIIT-swim. In the H-s rats, mitochondria delimited by their outer membranes were observed, and the inner membranes gave rise to the organization of the internal ultrastructure, with the mitochondrial ridges organized mostly in parallel and very close to each other, occupying the internal space of the mitochondrion. A phenotype with a more homogeneous electrodensity, similar to that of the C rats, was observed (Figure 8). These characteristics of density and ridge folding have been observed in the mitochondria of organs such as the heart because they have a high demand for ATP [44].

## 4. Discussion

HIIT is a training method that involves performing high-intensity exercise with sustained effort for periods ranging from a few seconds to a few minutes, followed by rest intervals [45]. This type of training has been shown to be safe and effective in improving fitness in general [46]; however, such training should be introduced gradually to ensure safety [47]. An exercise method such as HIIT may also be used to decrease risk factors for cardiovascular disease [48,49] as it can act as a protective agent for cardiomyocytes and blood vessels by decreasing inflammation in the heart and improving cardiac function [50]. This is especially important during childhood and adolescence due to the association with modifiable and preventive risk factors [1,5,6].

The Sprague Dawley rats in the H-s group that performed the 4-week HIIT-swim protocol were estimated to have started the protocol in their adolescent stage, with an approximate human-equivalent age of 12 years [51], and finished it at the beginning of adulthood. The 4-week training period was estimated to be equivalent to 8 years of exercise in humans [51]. This period is very important, as risk factors that present in childhood and adolescence [1,5,6] may have a significant impact in adulthood. Figures reported by WHO estimate that 80% of present-day adolescents do not maintain the minimum level of physical activity required for optimal health [10]. Therefore, the adolescents of today may become adults who are burdened with sedentary-sensitive morbidities, with increased risk factors for cardiovascular or other chronic noncommunicable diseases and resulting financial and social burdens [10].

Numerous changes have been observed in the ultrastructure of mitochondria that could correspond to adaptation processes and environmental requirements [52]. In the present study, in addition to observations of the ultrastructure of mitochondria using transmission electron microscopy, quantifications of the mitochondrial area, perimeter and circularity index were performed (Table 1). These morphometric parameters are important because changes in mitochondrial morphology influence cellular metabolic capacity [8,9]; consequently, these variations are important in cardiovascular physiology [53,54,55].

Regular exercise results in cardiovascular adaptations that protect the body by making it more resistant to chronic diseases [56]. In such adaptations, the mitochondrial network forms an essential part of the cardiac contractility process; this must maintain a mitochondrial structure that can produce the energy necessary to meet the demands of the heart [57]. However, the loss of mitochondrial control mechanisms caused by factors such as progressive aging, stress [19] or a sedentary lifestyle can lead to mitochondria with functional and structural abnormalities [19]. In the present study, it was observed that, in the S rats subjected to sedentary conditions, the internal organization of the mitochondria presented mitochondrial ridges with fragmentation at multiple points. These results suggested a possible functional impact, specifically a negative effect on the efficiency of the energy process [27]. A sedentary lifestyle also leads to lower exercise endurance and reduced cardiorespiratory capacity, which is known to decline when a sedentary state is acquired in adulthood [2]. A sedentary lifestyle could therefore influence the development of cardiac pathologies.

Other studies have highlighted characterizations of the mitochondrial ultrastructure in health- and age-related contexts. In aged and infarcted hearts, mitochondria with decreased numbers of membrane invaginations and abnormal organizations of mitochondrial crests with concentric arrangements have both been observed [52,58].

Researchers have also reported alterations in the mitochondrial homeostasis of cardiac tissue in patients with heart failure as conditioning adaptations to unfavorable environments and as indirect data of cardiac remodeling [59]. Mitochondrial ridges with altered organizations have also been reported. Transitions with ridge variations have been found to be age-dependent, and increases in curvature and fragmentation have also been associated with the progressive course of age [26]. In the present study, no such altered structural variations were observed, probably because rats in all study groups were young and without pathologies. However, some mitochondrial phenotypes similar to the previously described alterations were observed in the S rats; this may have been due to their sedentary lifestyle, which is a known risk factor with an impact on morphofunctional variations.

The loss of continuity in the inner membranes of S rats, along with their lack of parallel alignment and proximity, compared with the inner membranes of C rats suggests an adaptation in the mitochondrial ultrastructure that could correspond to the effect of the physiological course of a sedentary condition. These changes could indicate a decrease in the capacity to produce energy due to smaller surface areas of the inner membranes that make up the mitochondrial cristae [52] compared with C and H-s rats. Morphometric variations suggest an increase or decrease in the energy-generating capacity of mitochondria due to a direct relationship with the decrease or increase in the surface area of the inner mitochondrial membranes [42].

HIIT-swim induced effects on the physiological adaptation of conserved energy productivity in H-s, showing ultrastructural preservation when its phenotype was compared with S.

Therefore, when performing the protocol, the H-s rats were subject to the influence of an independent variable that induced adaptive mechanisms to respond to the energy demand required to perform the workouts. HIIT-swim was found to be a key factor in preserving an ultrastructure similar to that of a younger state (C). HIIT-swim might therefore be a protective factor for the preservation of inner membrane surfaces, as these are essential for energy production, which satisfies the high requirements of optimal heart function during the life cycle [15]. Such exercise is known to improve the efficiency of the mitochondrial network due to increased rates of mitochondrial biogenesis and the efficient elimination of dysfunctional or damaged mitochondria [60].

The results of the present study showed that the HIIT-swim group (H-s) showed morphological signs at the mitochondrial level of ultrastructure preservation compared with the non-training and sedentary group (S). In addition, the HIIT-swim protocol prevented age-related mitochondrial remodeling.

The strength and innovation of this research derives from its application of the HIIT-swim protocol in a heated pool for a period estimated to be equivalent to the human adolescent stage [51]. In addition, it involved three study groups (C, S, H-s), enabling an evaluation of ultrastructural remodeling in relation to HIIT-swim and age by means of transmission electron microscopy.

The present study may serve as a foundation for assessment of ultrastructural transitions and remodeling in additional age groups. Specifically, it may serve as a morphometric basis for studying the impact of HIIT on the structural preservation required for energy production of mitochondria in the heart. 

The techniques described in this paper might also be applied to modalities of different intensity. In future studies, dosing might be adapted for different groups of patients according to their physical condition. Other organs involved in energy metabolism might also be studied. 

A limitation of the present study was the small population of animals used (n = 18); however, the data did meet the conditions of a non-parametric longitudinal study, and revealed, in line with the findings of other authors, the benefit of exercise in relation to reducing the impact of prevalent noncommunicable diseases that affect the general population.

## 5. Conclusions

In the present study, the HIIT-swim protocol was found to have an impact on the morphometric parameters of H-s rats by significantly increasing mitochondrial areas and perimeters relative to those of S rats, suggesting a preservation of mitochondrial function in energy production.

The HIIT-swim protocol had the effect of preserving the typical structure of functionally healthy mitochondria with structurally organized membranes in H-s rats. It was able to prevent the mitochondrial remodeling and the functional impact observed in sedentary rats (S). These animals showed a loss of continuity in their mitochondrial cristae, suggesting a decrease in the ATP production necessary for the heart’s energy requirements.

In conclusion, further investigation is now warranted regarding morphofunctional variations in other age-range groups, involving adaptations of the HIIT-swim protocol, so that ultrastructural transitions of cardiac mitochondria may be better understood. The findings of the present study indicate that HIIT-swim may be an effective intervention for the prevention of cardiovascular diseases that are dependent on mitochondrial ultrastructural transitions associated with a sedentary lifestyle and age. Ultimately, HIIT-swim may decrease the economic effect on health care systems by preventing cardiovascular disease.

## Figures and Tables

**Table 1:** Mitochondrial morphometric parameters.

Parameters	Morphometric Parameters	C	S	H-s
Mitochondrial shape	Circularity index	0.738 ± 0.007	0.818 ± 0.006 *	0.837 ± 0.005 *
Mitochondrial size	Mitochondrial area (µm^2^)	0.262 ± 0.011	0.645 ± 0.024 *	0.761 ± 0.024 *#
	Perimeter (µm)	1.982 ± 0.045	2.992 ± 0.065 *	3.264 ± 0.054 *#

Summary of the results obtained for mitochondrial morphology variables: size (area and perimeter) and shape (circularity index). Values are expressed as mean ± standard error of the mean. *p* < 0.05 was considered statistically significant. * *p* < 0.05 vs. control group (C); # *p* < 0.05 vs. sedentary group (S).

## Data Availability

The data presented in this study are available on request from the corresponding author.
